# Palliative care professional education via video conference builds confidence to deliver palliative care in rural and remote locations

**DOI:** 10.1186/1472-6963-14-272

**Published:** 2014-06-19

**Authors:** Robin A Ray, Ofra Fried, Daniel Lindsay

**Affiliations:** 1 College of Medicine and Dentistry, Anton Breinl Research Centre for Health System Strengthening, James Cook University, Townsville 4811, Australia; 2 Townsville Health District Palliative Care Service, 100 Angus Smith Drive, Douglas 4814, Australia

**Keywords:** Rural workforce, Palliative care, Education, Video conferencing, Capacity, Confidence

## Abstract

**Background:**

People living in rural and remote locations are disadvantaged in accessing palliative care. This can be attributed to several factors including the role diversity and the low numbers of patients with specific conditions, as well as the difficulties rural health practitioners have in accessing opportunities for professional education. A program of multidisciplinary palliative care video conferences was presented to health practitioners across part of northern Australia in an effort to address this problem.

**Method:**

The educational content of the video conferences was developed from participant responses to an educational needs assessment. Following cycles of four consecutive video conferences, 101 participants completed evaluative on-line surveys. The quantitative data were analysed using frequencies and analysis of variance tests with post-hoc analyses where appropriate, and an accessibility and remoteness index was used to classify their practice location.

**Results:**

All participants found the content useful regardless of their remoteness from the tertiary centre, their years of experience caring for palliative care patients or the number of patients cared for each year. However, change in confidence to provide palliative care as a result of attending the video conferences was significant across all disciplines, regardless of location. Doctors, medical students and allied health professionals indicated the greatest change in confidence.

**Conclusions:**

The provision of professional education about palliative care issues via multidisciplinary video conferencing increased confidence among rural health practitioners, by meeting their identified need for topic and context specific education. This technology also enhanced the networking opportunities between practitioners, providing an avenue of ongoing professional support necessary for maintaining the health workforce in rural and remote areas. However, more attention should be directed to the diverse educational needs of allied health professionals.

## Background

Palliative care is an integral part of rural health care practice, but research about rural palliative care remains limited [1]. People living in rural and remote areas of Australia [2-4] and those from non-mainstream backgrounds, such as Indigenous Australians, particularly those living in remote areas [5] are disadvantaged in accessing palliative care. While some of this may be due to distance from urban specialist services, rural health care practitioners may also have limited experience and skills in the delivery of palliative care [2]. Robinson et al. [1] considered the most significant factor hindering the provision of high quality care in rural areas was lack of role preparation as most nursing and medical undergraduate courses do not cover this area of care adequately. They proposed that research was needed into ways of using and evaluating technology based educational innovations and consultations to support rural practitioners [1]. This paper reports quantitative evaluation findings from a series of video conferences designed to address the palliative care educational needs of doctors and other health care practitioners working at a distance from tertiary centres.

Recent migration patterns within Australia indicate that more people are moving to rural areas as part of the ‘tree-change’ or ‘amenity’ migration (moving to more desirable location for non-economic reasons), at rates of 1.5 percent in regional Queensland reversing the previous trend of rural population decline [6]. This is likely to result in an increased need for all health services including palliative care. The rural and remote context provides many positive aspects for palliative care. There is consistent evidence that many people prefer to be cared for at home at the end of life, and that this is particularly important to those living in rural and remote areas who otherwise, would experience isolation and distress if they had to travel into urban areas for care [4,7,8]. Local health care practitioners bring the added benefits for patients of longstanding care relationships, higher levels of community accountability and an understanding of local issues [4,9,10]. Role diversity is a core characteristic of rural practice, but in rural and remote areas of Australia, where the number of people per square kilometre ranges from 10–0.1 [11], the numbers of patients with specific conditions are not sufficient to enable primary care practitioners to maintain their skills [12]. Deficiencies in educational preparation for general practitioners delivering community palliative care also contribute to inadequacies in service provision [13,14].

Studies suggest that providing continuing education to local staff can improve rural palliative care [15,16] and reduce attrition rates in the rural workforce [17]. More importantly, rural practitioners value access to education and support as a means of enhancing the delivery of care in their communities [18]. However, rural general practitioners, who have an important role in providing palliative care in rural areas, acknowledge difficulties in maintaining knowledge and skills and accessing further education because of distance and the difficulty arranging locums [19-21]. Similarly, rural nurses consider that palliative care is integral to their role, but find it difficult in the absence of adequate support and continuing education [22,23]. Allied health practitioners working in rural areas also report being under-prepared and lacking access to continuing education [24,25]. The recommended educational strategies for these groups include conducting a needs analysis and designing context specific programs [3,26]. Local delivery of education which takes into account contextual factors can strengthen existing community expertise and commitment, enabling service providers to respond to community needs in a sustainable way [2]. With technological improvements and reduction in infrastructure costs during recent years, videoconferencing has become a widely accepted, cost effective medium for clinical consultations [27,28] and health care education in Australian and internationally [29-31]. The most commonly identified problems with videoconferencing relate to perceptions that the equipment is difficult to use and in educational situations, some participants feel less comfortable asking questions [30]. Additionally, videoconferencing has been rated as less effective than attending a meeting in person, but far preferable to commuting, particularly where distances were long [32]. However, video conferencing is useful in supporting health care professionals providing palliative care in rural or remote locations [33,34], thereby improving access to locally adapted palliative care for patients at the end of life [2,35].

### Aim

This study evaluated the educational impact of video conferencing to increase the confidence of doctors and other health professionals to deliver quality palliative care in rural and remote areas.

## Method

To undertake an effective evaluation of a series of educational sessions delivered to a wide geographical area, a mixed methods approach was adopted [36], ensuring that the qualitative component adhered to the RATS Guidelines on Qualitative Research. An analysis using both qualitative and quantitative data permitted the identification of specific enablers and impediments to success in delivering relevant palliative care education to practitioners in diverse locations.

This project was granted ethical clearance by the Townsville Health Service District Human Research Ethics Committee for the relevant Health Districts (HREC/10/QTHS/163) and undertaken in compliance with the Helsinki Declaration.

Rural health practitioners (n = 174) across north Queensland were recruited to the project through responding to information flyers and advertisements in health service and general practice newsletters, contact with local primary health care peak bodies, and ongoing information and reminder notices distributed through email contact lists generated during the project. Registered participants had the evaluation research explained to them and informed consent was obtained to assess their educational needs and participate in ongoing evaluative data. Consent was indicated by the participant’s return of the educational needs assessment (mostly through email) and their completion of the four monthly, on-line evaluation surveys. Emails and telephone calls were made to site contacts to remind participants about each video conference and evaluation cycle.

An educational needs assessment (ENA) survey was adapted from an existing tool [37] and piloted locally. Questions pertaining to United States of America law and practices were eliminated and other questions were reworded or added to address issues relevant to the local context and the needs of the study. The ENA included questions about previous palliative care related study; years of experience with palliative care patients, and locality data, as well as required levels of information by topic (see Additional file 1). ENAs were distributed by email to registered participants as they joined the programme. Level of knowledge (beginning or advanced) and topic ratings were used to develop the content for the video conferences. Other topics were added to the video conference schedule in response to suggestions from the on-line evaluations. Video conferences were delivered monthly by members of the tertiary level multidisciplinary palliative care team and a palliative care researcher, all experienced in working in rural and regional areas. The education session began with some lecture style input, interspersed with participant questions, followed by case based discussion and sharing of resources among all participants. Participant at remote sites were encouraged to provide case examples and share solutions from their own context. The time spent on each of these sections varied with the topic and the level of participant interaction. In most sessions, the structured input was provided by two to four members of the multidisciplinary palliative care team working as an expert panel. A list of the topics addressed can be seen in Table 1.

**Table 1:** Topics covered in palliative care video conferencing sessions

**Evaluation interval**	**Monthly topic**			
May to August	Transition to palliative care	Pain management	Ethical issues	Breaking bad news
September to December	Inpatient palliative care	End of life care	Discussing prognosis and advance care planning	Family care
February to May	Gut stuff	Palliative care emergencies	Community palliative care in rural and remote settings	Non-cancer palliative care
June to September	Managing MND and other related conditions	Living life fully: spirituality and sexuality	Altered mental states	Self care

An evaluative survey was developed using Survey Monkey software and piloted to determine the reliability of the items to generate appropriate responses. Validity could not be tested because participants will register a personal response to each topic related to their existing knowledge and experience. However, survey results correlate will with qualitative data suggesting an acceptable level of reliability. Four point continuum scales enabled participants to respond to questions about the topics covered in the previous four months. Statistical data generated were analysed using frequencies and analysis of variance (ANOVA) tests with post-hoc analyses where appropriate. Post-hoc analysis identified specific differences between subgroups and isolated where these differences lie [38]. The results of this statistical analysis informed the development of this paper.

Accessibility/Remoteness Index of Australia (ARIA) scores were used to categorise locations based on accessibility of places to goods and services and opportunities for social interaction [39]. Inner regional locations experience some restrictions to accessibility and opportunities for social interaction; outer regional locations are significantly restricted in both accessibility and social interaction; remote locations experience very restricted access and opportunities, while very remote locations have very little access to goods, services and opportunities for social interaction.

### Participants

A total of 101 participants completed this study, with 10 participants listing their profession as being a medical student or doctor, 71 identifying themselves as a nurse, 16 as an allied health professional (occupational therapist [5], physiotherapist [4], speech pathologist [2] or social worker [5]) and 4 in the ‘other’ category. Of the participants, 35 indicated that they worked in an inner regional area, 34 indicated they worked in an outer regional area, 28 said they worked in a remote location and 4 said they worked in a very remote location. Participant experience with palliative care patients can be seen below in Tables 2 and 3.

**Table 2:** Frequencies indicating participant’s year of experience with palliative care patients (N = 99)

5 or less	6-10	11-15	16-20	20+
5	15	9	17	53

**Table 3:** Frequencies indicating the numbers of palliative care patients cared for each year (N = 55)

5 or less	6-20	21-50	50+
10	20	10	15

## Results

### Content usefulness

On average, participants rated the content of the video conferencing programs as being useful for their work (M = 3.50, SD = 0.67). ANOVAs were conducted to analyse the effects of certain variables on ratings of content usefulness. The influence of profession on ratings of content usefulness was examined and the means and standard deviations for this analysis can be seen in Tables 2 and 3. Although no significant main effect of profession was found (*F*_(3,100)_ = 2.24, *p =* .088), post-hoc analyses revealed significant differences in ratings of content usefulness between allied health professionals and both medical doctors/students (*p =* .033) and nurses (*p =* .018). This suggests that allied health professionals found the content less useful for their work than did nurses and doctors (Table 4).

**Table 4:** The influence of profession on ratings of content usefulness

**Profession**	**N**	**M (SD)**
Medical Doctor/Student	10	3.70 (0.48)
Nurse	71	3.56 (0.60)
Allied Health Professional	16	3.13 (0.89)
Other	4	3.50 (1)

This table represents the participants’ responses to the statement: The content covered was useful for my work.

There were no significant differences in ratings of content usefulness based on practice location (*F*_(3,100)_ = 1.77, *p =* .158), suggesting that participants from all practice locations found the content equally useful. There were also no significant differences in ratings of content usefulness based on years worked in palliative care (*F*_(4,98)_ = .926, *p =* .453) or the number of palliative care patients cared for each year (*F*_(3,54)_ = 1.80, *p =* .159). These results indicate that all participants found the content of the video conferences useful regardless of their level of previous experience with palliative care patients.

### Confidence in palliative care topics before video conferences

ANOVAs were conducted to analyse the influence of profession and location of work on confidence in the palliative care topics prior to participating in the video conferences. For the purposes of this study, confidence is defined as a degree of assurance, being certain of an act or event; a marker for individual capacity, mental or physical ability to perform [40].

Medical students or doctors reported the highest confidence in palliative care topics (M = 3.06, SD = 0.52) followed by nurses (M = 2.89, SD = 0.55) and allied health professionals (M = 2.43, SD = 0.75). There was a significant difference in topic confidence before participating in a video conference based on profession (*F*_(2, 94)_ = 3.18, *p =* .028), with post-hoc analyses revealing that allied health professionals had significantly lower confidence in palliative care topics than both nurses (*p =* .008) and medical doctors or students (*p =* .013) before participating in the video conferences.

Participants from inner regional areas reported the lowest confidence in palliative care topics before participating in the videoconferences, with participants from very remote, outer regional and remote areas all reporting higher levels of confidence (see Table 5). There were significant differences between confidence levels based on location (*F*_(3,93)_ = 3.16, *p =* .028), with post-hoc analyses revealing that participants from inner regional (*p =* .003) and outer regional (*p =* .050) areas indicating significantly lower confidence in palliative care topics before participating in the videoconferences than those living in remote areas.

**Table 5:** Confidence before videoconferences and confidence change by location of work

**Work Location**	**Confidence before videoconferences**	**Confidence change**
	**M (SD)**	**M (SD)**
Inner regional	2.63 (.52)	.54 (.49)
Outer regional	2.79 (.69)	.60 (.43)
Remote	3.09 (.53)	.48 (.45)
Very remote	2.78 (.39)	.44 (.51)

### Confidence change

An average confidence change variable was created by subtracting participant’s confidence rating in the topic after the video conferencing session from their confidence in the topic before the session. As a result of this calculation, positive scores indicate an increase in confidence in the topic and higher scores indicate greater levels of confidence change.

Across all video conferencing sessions, there was a mean increase of confidence in the video conferencing topic of 0.54 (SD = 0.46) suggesting that the videoconferences were successful in increasing confidence in palliative care topics across all participants. On average, participants from inner regional, outer regional, remote and very remote areas indicated similar levels of confidence change after viewing the videoconferences (see Table 5). No significant difference in confidence change based on location of practice was found (*F*_(3,93)_ = .414, *p =* .743).

Participants who listed their profession as being a medical doctor or student (M = 0.65, SD = 0.37) reported the highest level of average confidence change in topics being presented in the video conferences. Allied health professionals (M = 0.58, SD = 0.42) reported the 2nd highest average confidence change, followed by nurses (M = 0.53, SD = 0.47) and the other category (M = 0.33, SD = 0.58). There was no significant difference in average confidence change based on profession (*F*_(3,93)_ = .422, *p =* .74).

The number of patients cared for each year had a significant influence on average confidence change as a result of attending video conferences (*F*_(3,53)_ = 4.60, *p =* .006). The relevant means and standard deviations for this analysis can be seen in Table 6. Participants who indicated caring for 5 or less palliative care patients had a significantly larger average confidence change than those participants who cared for 6 to 10, 11 to 20 and 21 to 30 palliative care patients each year. No other significant differences between groups were found. The amount of years working with people who had palliative care needs was found to have no significant influence on the amount of confidence change that participants reported (*F*_(4,91)_ = .761, *p =* .554).

**Table 6:** Confidence change by number of patients care for each year

**Number of patients cared for each year**	**Confidence change M (SD)**
5 or less	.98 (.48)
6-10	.47 (.44)
11-20	.57 (.45)
21-30	.36 (.32)

### Influence of palliative care training

ANOVAs were conducted to assess the influence of previous training in palliative care on confidence in palliative care topics before participating in video conferences and average confidence change as a result of participating in video conference sessions. The relevant means and standard deviations can be seen in Table 7. There was a significant difference found in confidence before participating in video conferences based on levels of education (*F*_(3, 92)_ = 12.01, *p* = .000). Post-hoc analyses revealed that those with no previous training had significantly lower confidence in palliative care topics before viewing video conferences than all other groups (all p’s < .05). Those who only had on-the-job training had significantly lower confidence in palliative care topics than those with post-graduate qualifications (*p =* .042) and short course training (*p =* .025).

**Table 7:** Influence of education on confidence and confidence change

**Education level**	**N**	**Confidence before videoconferences**	**Confidence change**
		**M (SD)**	**M (SD)**
Post-graduate	13	3.13 (0.35)	0.43 (0.39)
Short course	26	3.08 (0.51)	0.40 (0.43)
On-the-job	42	2.78 (0.52)	0.59 (0.46)
No previous training	12	2.10 (0.68)	0.80 (0.45)

An ANOVA was conducted to examine the influence of education levels on confidence change as a result of attending the videoconferences. As the results from this ANOVA were only marginally insignificant (*F*_(3, 92)_ = 2.54, *p* = .061), post-hoc analyses were conducted. These analyses indicated that those with no-previous training had significantly more change in confidence than those participants who had post-grad qualifications (*p =* .044) and short course training (*p =* .014).

## Discussion

The results of this study indicate that the locally generated content and ability to deliver repeated multidisciplinary educational opportunities to practitioners at their workplace enhanced the confidence of rural doctors, nurses and allied health professionals, to deliver quality palliative care. Confidence is a key aspect of the ability to deliver clinical care, and this study sought to improve the clinical capacity of rural health care providers to deliver palliative care locally. Confidence can be considered an important aspect of and a marker for professional capacity, as it encompasses the “skills, knowledge, confidence, job-readiness and ability to network and interact” that are required to achieve an outcome [41].

While years of experience and confidence show no correlation, practitioners working in areas where they care for lower numbers of palliative care patients reported the greatest increase in confidence as a result of participating the video conference sessions. This finding is important for not only for patient care, but also for rural workforce retention, as one of the major reasons health professionals leave rural and remote areas is the lack of professional support and access to educational opportunities [21]. The value of this form of continuing education is demonstrated in that just over half the participants [n = 53] had more than twenty years experience caring for people with palliative care needs, yet they still found the video conferences useful in their work and asked for the videoconference program to continue. This trend indicates an ongoing need for continuing professional education to enable practitioners from all disciplines to maintain and extend their generalist capabilities. This issue is further discussed elsewhere in a paper based on qualitative findings from the study.

Adopting a multidisciplinary approach to the delivery of this video conference series modeled multidisciplinary team work in palliative care practice. Complex issues inherent in palliative care were discussed in a sensitive manner by experienced practitioners both in the remote location and at the tertiary site. In this way, connections were made with the context in which the care was being delivered, while drawing on a range of professional opinion and expertise. Discussion that ensued between rural and remote practitioners and the multidisciplinary panel situated in the tertiary centre facilitated the development of professional networks, which could lead to more effective care [15]. However, while a multidisciplinary approach was adopted for many sessions, the content value by profession was variable.

Medical students rated amongst the highest level of confidence for topic pre video conference. This finding could reflect a misplaced judgment of their actual skills. Nevertheless, while the numbers of respondents are not statistically significant, this is an interesting finding considering the previous studies that suggest undergraduate education is lacking in palliative care content [1]. Recent availability of palliative care curriculum for undergraduate health care professionals funded by the Australian Government [42] and additional palliative care input to the medical curriculum by specialists in tertiary centers may have influenced this outcome. Medical doctors may report high levels of confidence before an education session because of the social expectations that they will be confident in their practice, assured, and certain of their ability to deliver best practice medicine [40]. However, despite the pre-video conference confidence by topic, medical doctors and medical students also reported the highest level of confidence change, thus demonstrating the efficacy of the educational content provided.

Nurses represent the largest sector of the health care workforce and deliver most of the formal palliative care [43]. Mirroring these figures, nurses accounted for the greatest numbers of participants (n = 71), frequently asking questions about specific cases. Contrary to expectations, nurses in remote areas indicated greater levels of confidence than those in inner areas, who might have better access to education and support. This may reflect the broad experience base of the rural and remote nursing workforce, which takes on additional tasks and responsibilities in the absence of medical support [23]. While nurses as a discipline reported higher levels of pre-video conference confidence than allied health, the change in nurses’ level of confidence was least among all disciplines. This could be attributed to the adequacy of nurses palliative care skills and knowledge prior to the video conferences [3] and the benefit received from the assurance that their practice is accurate and effective [40].

Allied health practitioners (occupational therapists, physiotherapists, speech pathologists and social workers) actively participated in the video conferences. Consistent with O’Toole’s findings [24] that allied health practitioners were under prepared for rural work, they reported the lowest levels of confidence before the video conferences. Despite rating the content as less useful, allied health practitioners recorded the second highest level of confidence change. These results indicate that while the content was perceived as being not as relevant, allied health practitioners did gain information that increased their confidence in the practice of palliative care [3]. However, the diversity of allied health practice, the geographical distances between patients in rural and remote settings together with the limited number of allied health practitioners in rural areas increases the complexity of meeting the educational needs of this group in a multidisciplinary one hour per month video conference. Allied health professionals responding to the education needs assessment would have been proportionally less, thus under representing their needs for specific topics or perspectives on said topics. Future video conference sessions need to focus on allied health practice in palliative care paying more attention to the range of educational needs across the spectrum of allied health.

The strong correlation between those who had no previous training or some on-the -job training and the significant rise in confidence after the video conference series is an important factor for rural workforce planning. Health care professionals who have moved from metropolitan centers to rural areas for lifestyle reasons or partner employment reasons [44], or who have had little educational preparation for providing palliative care, may be ill equipped for the generalist roles required in rural practice. This, coupled with the often transient nature of the workforce in outer rural and remote areas, underscores the need for educational programs relevant to the local care context [3]. While large variations in pre and post confidence levels across disciplines indicates that the video conferencing program is fulfilling an educational need, it also suggests that palliative care topics may need to be presented regularly, addressing varying levels of complexity and the diverse skills required of a multidisciplinary team.

### Limitations

The findings from this study cannot be generalized as the sample was self selected and limited to those participants who had work email addresses, access to computers or were willing to take the time to complete the survey. However, the correlations that are presented are statistically significant and further qualitative data reported elsewhere confirms the usefulness of these sessions for rural and remote practitioners.

## Conclusions

The provision of professional education about palliative care issues via multidisciplinary video conferencing successfully met a significant educational need for rural health care professionals. It provided regular opportunities for educational input and professional support, essential to maintaining their practice confidence in providing locally adapted palliative care. Video conferencing enabled discussion of complex, often sensitive issues relevant to their caseload without having to leave their rural or remote work location. Content derived from participants expressed needs was generally well accepted. However, more attention should be directed to the diverse educational needs of allied health professionals. Using this technology enhanced the networking opportunities between rural practitioners and with the tertiary facility, providing an avenue of ongoing professional support necessary for maintaining the health workforce in rural and remote areas. While this study has demonstrated the educational value of regular video conferencing to support practitioners delivering palliative care, further research is required to explore patient and family satisfaction, and to measure improvements to palliative care services delivered in rural and remote areas.

## Competing interests

The authors declare that they have no completing interests.

## Author’s contributions

RR developed the evaluation project including the online survey and applied for the grant to support a research assistant, contributed to the education sessions, managed the data collection and analysis, developed the initial draft of the paper and completed the final paper. OF contributed to the development of the project, managed and contributed to the education sessions, contributed to the literature review, revised the first draft of the paper. DL analysed the quantitative data, prepared the results section of the paper and contributed to the interpretation of the data in the discussion section. All authors read and approved the final manuscript.

## Authors’ information

RR Has worked with people needing palliative care in rural areas, currently a social health researcher with interests in non-cancer palliative care and rural workforce recruitment.

OF Palliative care medical staff specialist who has been working in rural and regional palliative care since 1998.

DL Completing a PhD in psychology with interests in cognitive processes related to health risk behaviour.

## Pre-publication history

The pre-publication history for this paper can be accessed here:

http://www.biomedcentral.com/1472-6963/14/272/prepub

## Supplementary Material

Additional file 1 Educational needs assessment survey. Click here for file
