# Supplementary material for: Palliative care professional education via video conference builds confidence to deliver palliative care in rural and remote locations
Source: BMC Health Serv Res. 2014 Jun 19;14:272. doi: 10.1186/1472-6963-14-272 (PMC4085715; doi:10.1186/1472-6963-14-272)
Supplement: Additional file 1 — Educational needs assessment survey. [file 1472-6963-14-272-S1.docx]

**Appendix A**

**Educational Needs Assessment Survey**

**What level of information do you feel you need to manage these common clinical situations?**

**Please indicate using the following codes.**

1. **= Introductory information**
2. **= Extend your current knowledge**
3. **= Not applicable in my work**

| **No** | **Patient/family interactions and clinical management** | **1** | **2** | **3** |
| --- | --- | --- | --- | --- |
| 1 | Conducting a family conference to discuss goal setting |  |  |  |
| 2 | Giving “bad news” to a patient or family |  |  |  |
| 3 | Assessing patient decision-making capacity |  |  |  |
| 4 | Discussing advance care planning with patients and families |  |  |  |
| 5 | Discussing treatment withdrawal (e.g., antibiotics, hydration, ventilator) |  |  |  |
| 6 | Managing requests for futile treatments |  |  |  |
| 7 | Discussing DNR orders and advance health directives |  |  |  |
| 8 | Identifying when a patient needs a palliative approach to care |  |  |  |
| 9 | Discussing a shift in treatment approach from curative to comfort care |  |  |  |
| 10 | Discussing making a referral to a specialist Palliative Care service |  |  |  |
| 11 | Recognising dying |  |  |  |
| 12 | Managing end-of-life care |  |  |  |
| 13 | Assessing pain |  |  |  |
| 14 | Prescribing oral opioid analgesics |  |  |  |
| 15 | Prescribing parenteral opioid analgesics |  |  |  |
| 16 | Converting from one opioid to another |  |  |  |
| 17 | Managing common opioid side effects (sedation, confusion, nausea, constipation) |  |  |  |
| 18 | Using adjuvant analgesics (e.g., tricyclics, steroids, anticonvulsants) |  |  |  |
| 19 | Assessing and managing delirium |  |  |  |
| 20 | Assessing and managing dyspnoea |  |  |  |
| 21 | Assessing and managing nausea/vomiting |  |  |  |
| 22 | Assessing and managing constipation |  |  |  |

**Please indicate which of the following topics you would like included in the video conference program, using the following codes:**

1. **= Yes**
2. **= No**

| **No** | **Topic** | **1** | **2** |
| --- | --- | --- | --- |
| 1 | Pain assessment and management |  |  |
| 2 | Assessment and management of nausea and vomiting |  |  |
| 3 | Assessment and management of delirium |  |  |
| 4 | Assessment and management of dyspnoea |  |  |
| 5 | Assessment and management of constipation |  |  |
| 6 | Giving “bad news” |  |  |
| 7 | Running a family conference |  |  |
| 8 | Discussing prognosis |  |  |
| 9 | Introducing and facilitating advance care planning |  |  |
| 10 | Managing requests for futile treatments |  |  |
| 11 | Managing palliative care patients in the community |  |  |
| 12 | Inpatient palliative care: the who, why, when and where |  |  |
| 11 | Ethics: DNR orders, advance directives, decision making capacity |  |  |
| 12 | Use of intravenous hydration and/or artificial feedings in palliative care |  |  |
| 13 | Spirituality in palliative care: why is it important and what is the health practitioner’s role? |  |  |
| 14 | Incorporating and supporting family caregivers |  |  |
| 15 | End-of-life care |  |  |
| 16 | Recognising and managing dying |  |  |
| 17 | Sexuality in palliative care |  |  |
| 18 | Self care |  |  |
| 19 | Other (please specify) | | |

**About your practice**

| **On average how many palliative care patients would you care for each year? _____** |
| --- |

| **When caring for palliative care patients how frequently do the following events occur?** | **Weekly or more** | **Every few weeks** | **Every few months** | **Rarely** | **Never** |
| --- | --- | --- | --- | --- | --- |
| Provide home/community care |  |  |  |  |  |
| Hospital admission for symptom control |  |  |  |  |  |
| Hospital admission for respite care |  |  |  |  |  |
| Referral of patients to a Palliative Care specialist |  |  |  |  |  |

**This is the end of the survey thank you for your time**

Source. Education Needs Assessment Survey adapted with permission from the Educational Needs Assessment, pp 45-47 in:

Centre to Advance Palliative Care, Policies and Tools for Hospital Palliative Care Programs: A Crosswalk of National Quality Forum Preferred Practices. (New York: Centre to Advance Palliative Care, 2008), www.capc.org<http://www.capc.org>.
